# Isolation and Characterization of Nine Microsatellite Loci for a Parasitoid Wasp, *Encarsia smithi* (Silvestri) (Hymenoptera: Aphelinidae)

**DOI:** 10.3390/ijms14010527

**Published:** 2012-12-27

**Authors:** Ryuji Uesugi, Yasushi Sato

**Affiliations:** Tea Pest Management Research Team, Department of Tea, National Institute of Vegetable and Tea Science, Kanaya, Shimada, Shizuoka 428-8501, Japan; E-Mail: lucifer@affrc.go.jp

**Keywords:** genetic marker, genetic diversity, microsatellites, spiny whitefly, parasitoid wasp, classical biological control

## Abstract

The parasitoid wasp *Encarsia smithi* is an important agent in the classical biological control of two species of invasive spiny whiteflies, *Aleurocanthus spiniferus* and *Aleurocanthus camelliae*. To evaluate the performance of parasitism indexed by genetic diversity, a highly polymorphic genetic marker is required. In this report, nine microsatellite loci are described for *E. smithi*. The microsatellite loci were obtained through the construction of an enriched library and exhibited polymorphisms (2–6 alleles per locus) and high levels of expected heterozygosities (0.203–0.780, average 0.537). Linkage disequilibrium and null alleles were not detected in these microsatellite loci. The isolated microsatellite markers may be useful to estimate the genetic diversity of *E. smithi*.

## 1. Introduction

The parasitoid wasp *Encarsia smithi* (Silvestri) (Hymenoptera: Aphelinidae) is an important agent in the classical biological control of the orange spiny whitefly *Aleurocanthus spiniferus*, which is an invasive pest of citrus trees in Japan, the Caribbean islands, Micronesia, Hawaii, southern Africa, and Italy. Because *E. smithi* has such a strong ability to depress the population density of the whitefly, breeding and release programs of the wasp have often been implemented in *Citrus* orchards [1–4]. In addition, *E. smithi* plays an important role in the control of the tea spiny whitefly *Aleurocanthus camelliae*, which is a relatively new invasive pest of tea in China, Taiwan, and Japan. The Japanese population of *E. smithi* in citrus orchards originated from only 20 individuals collected from China in 1925 [5]; therefore, this species has experienced a strong population bottleneck. If *E. smithi* has lost significant genetic diversity and population fitness, releasing individuals obtained from native ranges to maximize genetic variation may improve their ability to parasitize in Japan [6]. Molecular markers have been useful to evaluate the genetic diversity correlated with population fitness [7]. We herein report the isolation and characterization of nine new microsatellite loci useful for estimating genetic diversity of *E. smithi*.

## 2. Results and Discussion

Forty-four sequences of 48 that originated from the enriched microsatellite library of *E. smithi* were unique, and 36 contained microsatellite repeats. Nine polymerase chain reaction (PCR) primer pairs of 16 designed successfully amplified putative target regions and showed polymorphisms in 32 individuals. These isolated microsatellites have simple repeat motifs and are easy to amplify using a low number of PCR cycles. The numbers of alleles per locus ranged from two to six alleles (average 3.78), and the expected heterozygosities ranged from 0.203 to 0.780 (average 0.537) (Table 1). Significant deviation from Hardy-Weinberg equilibrium (*p* < 0.05) and significant linkage disequilibrium (*p* < 0.05) were not detected in any loci. In addition, analysis performed with MICRO-CHECKER software showed no significant evidence for null alleles (*p* < 0.05). Consequently, the microsatellites had substantial polymorphisms and high independency and did not have detectable null alleles, indicating their usefulness in estimation studies of population genetic.

## 3. Experimental Section

We collected 50 individuals from parasitizing pupae of *A. spiniferus* found in an orchard of *Citrus unshiu* at Okabe, Fujieda City, Shizuoka Prefecture, Japan (34°55′45.88″N, 138°16′29.40″E) on 22 April 2010. These individuals were preserved in 99.5% ethanol at −20 °C. Genomic DNA was extracted using the GenomicPrep Cells and Tissue DNA Isolation Kit (GE Healthcare UK, Little Chalfont, Buckinghamshire, UK). DNA was digested using the restriction enzyme *Sau*3AI (TaKaRa Bio, Shiga, Japan). The resulting fragments were ligated into *Sau*3AI cassettes (TaKaRa Bio) and amplified using a cassette primer (TaKaRa Bio) by PCR. Fragments ranging from 300 to 1000 bp were hybridized to 5′ biotin-labeled oligonucleotide probes (biotin-(CA)_15_) after denaturation. The DNA molecules bound to the probes were isolated by means of Streptavidin MagneSphere Paramagnetic Particles (Promega, Madison, WI, USA). After rinsing the particles four times in washing buffer (0.1 × SSC), selected DNA fragments were eluted by 250 μL of ultrapure water. The resulting DNA fragments were amplified by PCR and digested with *Sau*3AI (TaKaRa Bio) to remove the cassettes. These microsatellite-enriched fragments were ligated into pUC118/*Bam*HI (TaKaRa Bio) and cloned into *Escherichia coli* DH5α-competent cells (TaKaRa Bio). Plasmids from these clones were prepared with the illustra plasmidPrep Mini Spin Kit (GE Healthcare UK). The plasmids of the 48 clones were sequenced using the BigDye Terminator Cycle Sequencing Kit version 3.1 (Applied Biosystems, Foster City, CA, USA) and the ABI PRISM 310 Genetic Analyzer (Applied Biosystems). PCR primers for the sequences, including the microsatellites, were designed with Primer3plus software [8].

Levels of locus polymorphism were assessed in 32 individuals of *E. smithi* collected in an orchard of *C. unshiu* at Okabe, Fujieda City, Shizuoka Prefecture, Japan (34°55′45.88″N, 138°16′29.40″E) on 22 April 2010, which originated from the same population used for constructing the genomic library. PCR amplifications were performed using the GeneAmp PCR System 9700 (Applied Biosystems) under the following conditions: 4 min at 94 °C followed by 26–33 cycles of 30 s at 94 °C, 30 s at 55 °C or 58 °C (annealing temperature), and 1 min at 72 °C, followed by 10 min at 72 °C (Table 1). A total reaction volume of 20 μL was used containing 0.4 μM of each primer, 0.25 mM of dNTPs, 10 mM Tris-HCl (pH 8.4), 50 mM KCl, 1.5 mM MgCl_2_, 1 U of DNA polymerase (TaKaRa Ex *Taq*), and 1 μL of the template DNA solution (about 1 ng/μL) prepared by the method of Osakabe *et al.* [9]. The primers that successfully amplified the template DNA solution were labeled with 6-FAM, NED, or HEX fluorescent dyes. PCR products from these labeled primer pairs were electrophoresed along with GeneScan 400HD (ROX) size standard (Applied Biosystems) on an ABI PRISM 310 Genetic Analyzer (Applied Biosystems).

Levels of genetic diversity (expected heterozygosity) and fixation indices for each locus were computed using Fstat version 2.9.3.2 [10]. Deviation from Hardy-Weinberg equilibrium for each locus and linkage disequilibrium for each pair of loci was tested using the randomization method after Bonferroni correction using Fstat version 2.9.3.2 [10]. The presence of null alleles was tested using Micro-Checker version 2.2.3 [11].

## 4. Conclusions

This is the first report on the isolation of microsatellite loci in *Encarsia* species, with the exception of the use of ISSR-PCR in *E. diaspidicola* and *E. berlesei* [12]. The characterized microsatellite markers are well suited to estimate the genetic diversity of *E. smithi* populations and will improve the classical biological control of orange spiny whitefly and tea spiny whitefly using *E. smithi*.

## Figures and Tables

**Table 1:** Primer sequences and characteristics of nine polymorphic microsatellite loci in 32 individuals of *Encarsia smithi*.

Locus Designation (Accession No.)	Primer Sequence (5′-3′) a	Annealing Templature	No. of PCR Cycles	Repeat Motif	Sequence Size (bp)	Size Range of Allele (bp)	*NA*^b^	*H_O_*^c^	*H*_E_^d^	*F*^e^
*Es002* (AB715240)	F: GAGGATTCAATTCGCACCTA R: GTAGCCCGCCAATAAATTAC	58 °C	26	(CA)_12_	201	201–221	5	0.438	0.490	0.107
*Es011* (AB715241)	F: CGACACGAACAACCCTAAAC R: GCGAAGAGACACGACTTGAT	55 °C	29	(GT)_12_	217	217–237	2	0.375	0.308	−0.216
*Es014* (AB715242)	F: TATCACCAGGAGCATCATCA R: GGAGCCTCTTATCGGTCTCT	58 °C	33	(CA)_11_	251	249–283	6	0.720	0.759	0.052
*Es051* (AB715243)	F: TCGTAAAATTATGCGAGCTG R: GATCCGAGTGGTACTGTGCT	58 °C	31	(GT)_10_	130	128–130	2	0.531	0.503	−0.056
*Es056* (AB715244)	F: ACCGTGTATCATCCGTTGAC R: GTGCGTGCGAGTATCTTCC	58 °C	31	(GT)_9_	183	180–185	5	0.750	0.780	0.039
*Es060* (AB715245)	F: TGCAACGTCTTCAAGTCCAG R: ATGCACGTCAATTTGTTTCG	58 °C	33	(GT)_9_	183	183–193	4	0.218	0.203	−0.077
*Es064* (AB715246)	F: CGAACCGATAAGAAGCCTGT R: CGTACACGCAACAATCGAAG	55 °C	26	(GT)_10_	155	143–155	3	0.687	0.555	−0.239
*Es082* (AB715247)	F: CAATAATTCCACCCAGCAC R: TGTTAAACACGGTGGAAAGAG	55 °C	28	(CA)_15_	126	104–126	5	0.749	0.729	−0.028
*Es083* (AB715248)	F: ATCCTCCGCGTTAGTACATC R: CATGACTACACACCCACGTC	55 °C	26	(GT)_9_	131	131–139	2	0.312	0.503	0.379

a F, forward primer; R, reverse primer.

b Number of alleles.

c Observed heterozygosity.

d Expected heterozygosity.

e Fixation index.
